# GDPF: a data resource for the distribution of prokaryotic protein families across the global biosphere

**DOI:** 10.1093/nar/gkad869

**Published:** 2023-10-12

**Authors:** Zhuo Pan, Dan-dan Li, Peng Li, Yu Geng, Yiru Jiang, Ya Liu, Yue-zhong Li, Zheng Zhang

**Affiliations:** State Key Laboratory of Microbial Technology, Institute of Microbial Technology, Shandong University, Qingdao 266237, China; Institute of Marine Science and Technology, Shandong University, Qingdao 266237, China; State Key Laboratory of Microbial Technology, Institute of Microbial Technology, Shandong University, Qingdao 266237, China; State Key Laboratory of Microbial Technology, Institute of Microbial Technology, Shandong University, Qingdao 266237, China; State Key Laboratory of Microbial Technology, Institute of Microbial Technology, Shandong University, Qingdao 266237, China; State Key Laboratory of Microbial Technology, Institute of Microbial Technology, Shandong University, Qingdao 266237, China; Suzhou Research Institute, Shandong University, Suzhou 215123, China; State Key Laboratory of Microbial Technology, Institute of Microbial Technology, Shandong University, Qingdao 266237, China; State Key Laboratory of Microbial Technology, Institute of Microbial Technology, Shandong University, Qingdao 266237, China

## Abstract

Microorganisms encode most of the functions of life on Earth. However, conventional research has primarily focused on specific environments such as humans, soil and oceans, leaving the distribution of functional families throughout the global biosphere poorly comprehended. Here, we present the database of the global distribution of prokaryotic protein families (GDPF, http://bioinfo.qd.sdu.edu.cn/GDPF/), a data resource on the distribution of functional families across the global biosphere. GDPF provides global distribution information for 36 334 protein families, 19 734 superfamilies and 12 089 KEGG (Kyoto Encyclopedia of Genes and Genomes) orthologs from multiple source databases, covering typical environments such as soil, oceans, animals, plants and sediments. Users can browse, search and download the distribution data of each entry in 10 000 global microbial communities, as well as conduct comparative analysis of distribution disparities among multiple entries across various environments. The GDPF data resource contributes to uncovering the geographical distribution patterns, key influencing factors and macroecological principles of microbial functions at a global level, thereby promoting research in Earth ecology and human health.

## Graphical Abstract

**Graphical Abstract:**
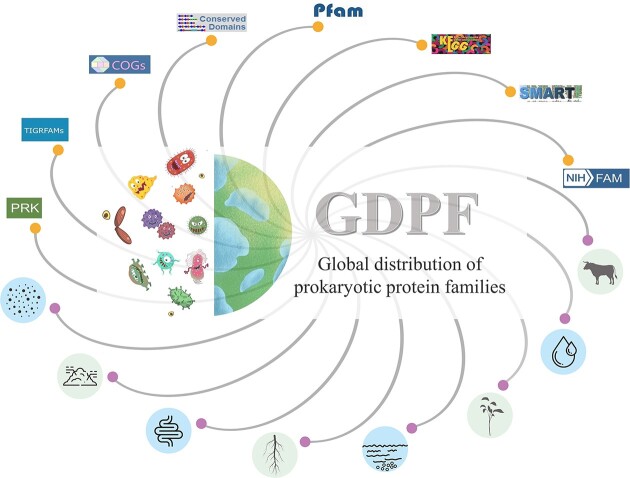

## Introduction

Microorganisms are ubiquitous on Earth, hidden on people’s skin, gut and soil, rivers, oceans and other environments, and their functions constitute pivotal factors influencing human health, disease development and global ecological changes (1,2). These microorganisms form intricate communities that drive nearly all recognized ecological processes within ecosystems, acting as vital links that connect material cycles and energy flow across different spheres, including the atmosphere, hydrosphere, lithosphere and biosphere (3). Until now, the vast majority of microbial biogeography research has concentrated on uncovering the phylogenetic diversity of microorganisms in complex environments, disregarding their functional diversity; nevertheless, it is the functions demonstrated by these microorganisms that genuinely propel the relevant ecological processes (4). Therefore, understanding the distribution of microbial functional characteristics in the environment will significantly contribute to a deeper comprehension of the relationship between microbial communities and natural geography from a functional perspective.

The functional potential of microorganisms relies on the collection of capabilities encoded by the genes included in their genomes (5,6). Many coexisting but taxonomically distinct microorganisms can encode the same metabolic functions, leading to limited insights provided by taxonomic approaches into the role of microbial communities in ecosystem functioning (7). It has been widely advocated to directly study the gene content of microbial communities and treat genes as potential functional traits of microorganisms (5,7). The functional annotation of microbial genes largely relies on their family classification (8,9). Members of the same family descending from a common ancestor usually have similar biochemical functions, three-dimensional structures and significant sequence similarities (10). Multiple families can be grouped into larger homologous superfamilies, which may lack identifiable sequence homology but still retain structural and mechanistic similarities (11). Numerous studies have quantified the functional aspects of microbial communities in specific environments (e.g. the human gut) based on families (12–16). However, comprehensive data on the distribution of microbial functional families in the global biosphere are still lacking.

Our previous research revealed that the proportion of sequenced genomes of global microbial communities has reached a high level (17). Mapping sequenced genome information to communities has become a crucial approach for studying microbial community functions (18–22). Here, we considered the global different environments as a unified system and leveraged data from the Earth Microbiome Project (EMP) along with sequenced genome information to construct the GDPF database (global distribution of prokaryotic protein families, http://bioinfo.qd.sdu.edu.cn/GDPF/), covering 10 000 microbial communities from environments such as soil, oceans, animals, plants and sediments. GDPF provides global distribution information for 36 334 protein families, 19 734 superfamilies and 12 089 orthologs encoded by prokaryotic genes from multiple source databases. Users can access the relative abundance, occurrence frequency and distribution data of these entries in various environments, thereby enabling easy comparison of their distribution differences across multiple environments to facilitate functional research.

## Data collection and database construction

### Data collection of microbial communities

The EMP was a systematic attempt aimed at sampling the Earth’s microbial communities on an unparalleled scale to characterize global microbial taxonomic and functional diversity (1). Our analyses were carried out using a subset of 10 000 samples released by the EMP, which serve as representatives of diverse environmental types. A modest sequencing depth of 5000 observations was randomly chosen for each sample. By employing the Deblur analysis method (23), we obtained a total of 262 011 amplicon sequence variants (ASVs) from the 10 000 samples.

### Mapping ASVs to genomes

**Figure 1.**
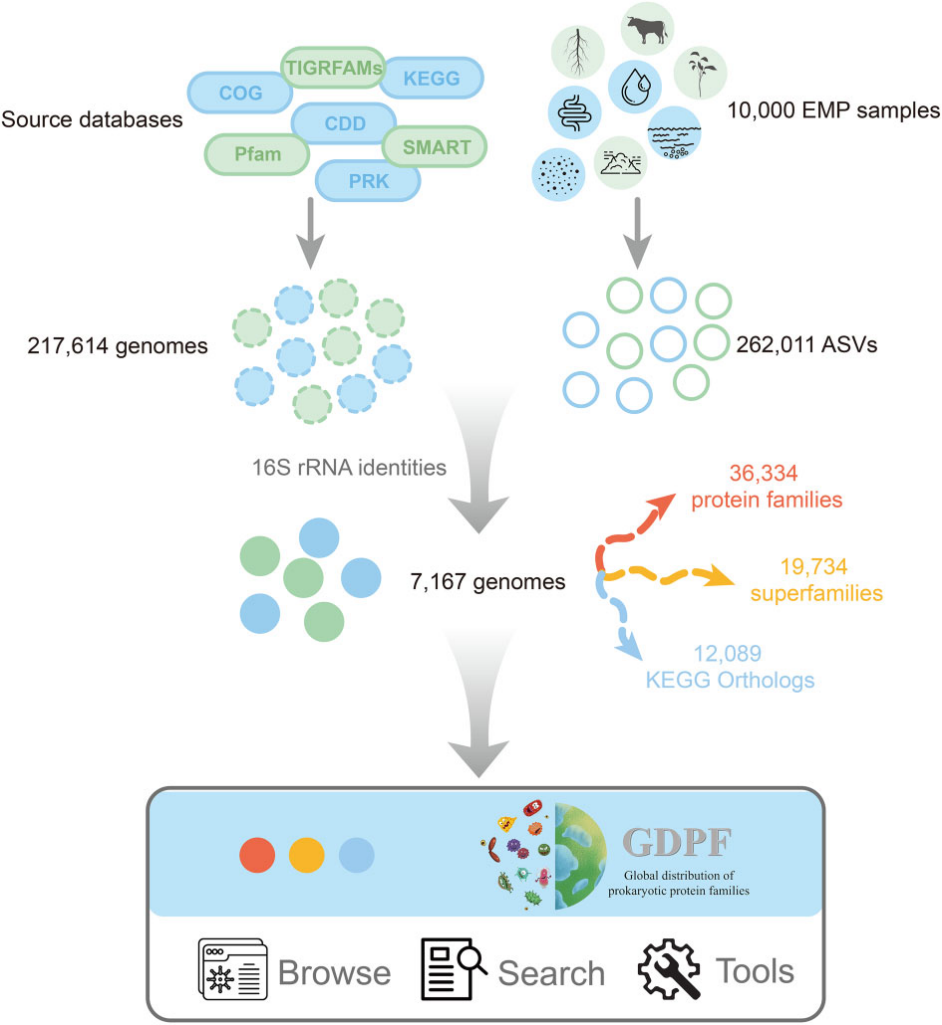
Workflow of GDPF database construction.

We gathered the genome information for all 217 614 bacteria and archaea available in the NCBI Reference Sequence database (24). Subsequently, we mapped the 16S tags from the EMP data to those derived from these genomes, using identity thresholds of >97%, 98.6% or 100%, along with 95% alignment coverage (Figure 1). The 100% identity threshold represents the most stringent and accurate match, while the 98.6% and 97% thresholds correspond to the new and traditional criteria for species definitions, respectively (25). The 100% threshold facilitates distinguishing closely related microorganisms, the 97% threshold allows for broader mapping to related microorganisms and the 98.6% threshold serves as a compromise between the two. In cases where multiple genomes were discovered, we selected those with the highest alignment identities and lengths. Ultimately, we mapped a total of 7167 genomes.

### Environmental classification

In a hierarchical framework, EMP ontology (EMPO) is utilized to categorize samples to different environments, capturing the primary dimensions of microbial community diversity (1). The classification system includes 17 microbial environments at level 3 (EMPO_3), which is divided into free-living and host-associated environments at level 1 (EMPO_1), and further distinguished between saline and non-saline environments (for free-living) and animal and plant environments (for host-associated) at level 2 (EMPO_2). The 10 000 samples released by the EMP aim to give equal (as possible) representation across various EMPO_3 environments (Supplementary Table S1).

### Annotation of functional families

For the obtained microbial genome-encoded protein sequences, we employed tools such as RPS-BLAST, eggNOG and BlastKOALA to perform functional annotation and family classification (11,26,27). This process involves multiple widely used protein classification databases, each having their own field of interest (e.g. superfamilies, orthologous groups, functional and structural domains). The Conserved Domain Database (CDD) is a resource for annotating functional units of proteins that utilizes 3D structures to provide insights into sequence–structure–function relationships and organizes conserved domains from multiple source databases into nonredundant superfamilies (11). The Protein families (Pfam) database is an extensive collection of multiple sequence alignments and hidden Markov models, covering the majority of common protein domains and families (10). The Kyoto Encyclopedia of Genes and Genomes (KEGG) establishes connections from collective sets of genes in the genome to high-level functions of the cell and the organism, while the KEGG Orthology (KO) database is used to directly associate genes with advanced functions and can be linked to the Gene Ontology (GO) knowledgebase (9,28). The Clusters of Orthologous Groups of proteins (COG) is a popular tool for functional and comparative genomics of bacteria and archaea, enabling precise functional assignments for proteins from different genomes (8). The Simple Modular Architecture Research Tool (SMART) provides a platform for comparative research on complex domains within genes and proteins (29). The PRotein K(c)lusters (PRK) consist of reference sequence proteins encoded by complete prokaryotic and chloroplast plasmids and genomes (30). The Institute for Genomic Research’s database of protein families (TIGRFAMs) offers a tool for identifying functionally related proteins based on sequence homology and has been incorporated into NCBIfam (31).

### Global distribution of functional families

Based on the mapping relationship between genomes and ASVs, we analyzed the global distribution of families, superfamilies and KOs using the EMPO environmental classification systems (Figure 1). First, we calculated the copy numbers of each entry (family, superfamily or KO) in each genome, normalized by the number of 16S ribosomal RNA (rRNA) genes encoded by the genome. Subsequently, for each EMP sample, the relative abundance of each entry was computed based on the sequence abundance of the ASVs mapped to the genomes. The relative abundance is expressed as the genes/cell value, the average number of genes for that entry carried by each cell in the community. As the mapping of ASVs to genomes was determined using three different thresholds (97%, 98.6% and 100%), three distinct relative abundances were also calculated for each entry. The relative abundance of each entry in 10 000 microbial communities was displayed on a world map and subjected to statistical analysis and comparisons based on EMPO_3 environments. Moreover, we also calculated the occurrence frequency of individual entry in each EMPO_3 environment (measured as the number of communities where the entry was detected divided by the total number of communities). The global distribution maps of entries were mainly constructed using the ‘ggplot2’ and ‘maps’ packages in R.

### Database construction

The GDPF database was built on the Java Virtual Machine and Java Application Programming Interface, along with Vue 2.x, and it ran on the Apache Tomcat server. The MySQL relational database was responsible for storing and managing all data in the database. The server-backend development was based on the JAVA language, while the web-frontend interface was implemented using Hyper Text Markup Language, JavaScript and Cascading Style Sheets. The frontend and backend interacted using the JAVA language, enabling the implementation of features such as search and submission to enhance response time and accelerate browsing speed. Software development tools include Python 3.9.13, Pandas 1.4.3, Numpy 1.21.6, Matplotlib 3.6.1 and Seaborn 1.21.6. The GDPF database can be accessed online without registration and is recommended for use on the Chrome browser.

## Database content and usage

### Data summary

**Figure 2.**
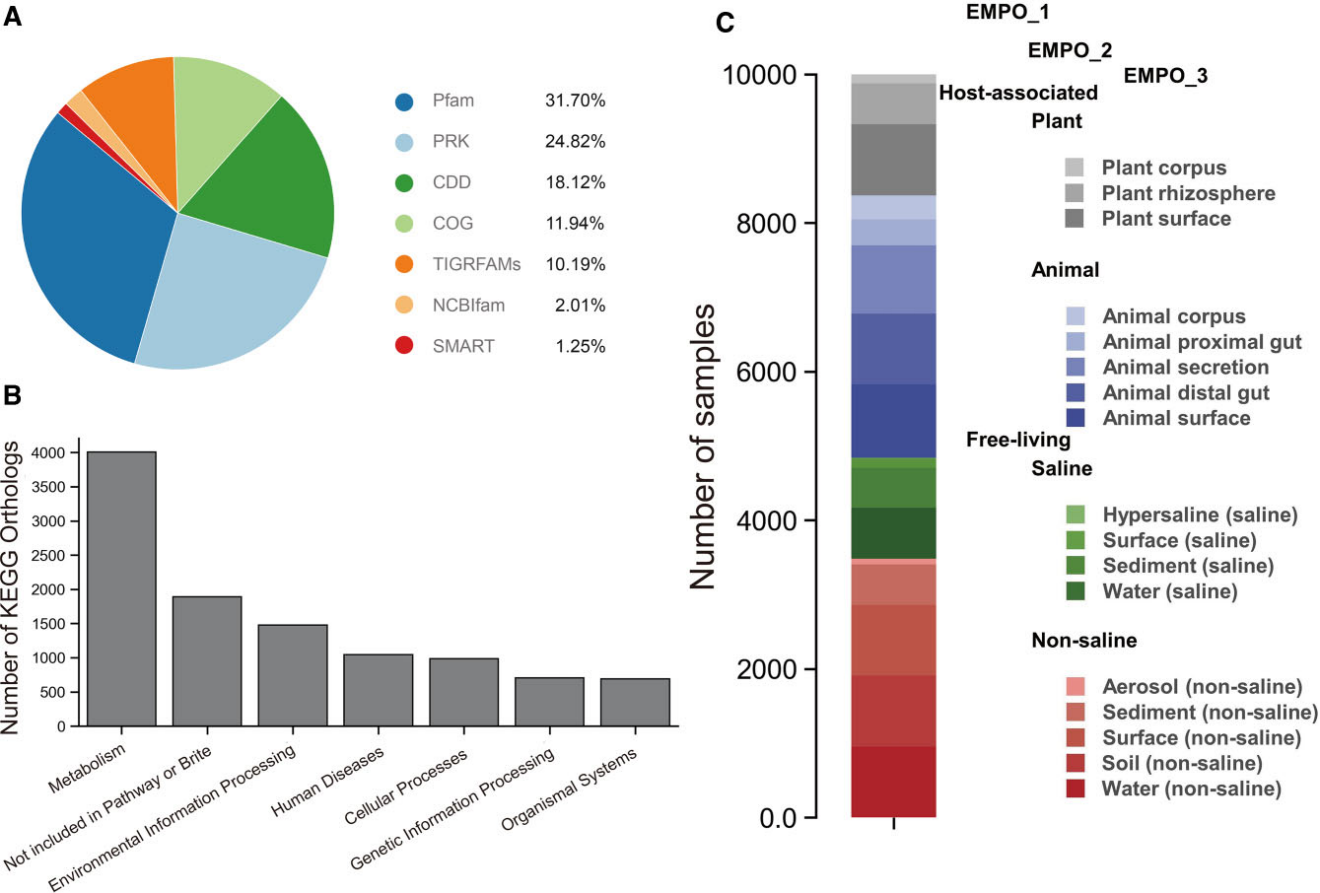
Data summary of the GDPF database. (**A**) Proportion of protein families from multiple source databases. (**B**) Quantities of annotated KOs with different KEGG pathways (level 1). (**C**) Environmental classification system employed by the database.

The GDPF database currently offers global distribution information for 36 334 protein families from the classifications of CDD, Pfam, SMART, COG, PRK and TIGRFAMs (Figure 2A). Among them, the number of protein families derived from Pfam is the largest (11 503), followed by PRK (9018) and CDD (6584). These protein families are further clustered into 19 734 superfamilies, where each superfamily represents a group of conserved protein structural models that are highly correlated in evolution.

GDPF also includes 12 089 KOs, along with the corresponding KEGG pathways for each KO, representing their potential involvement in various physiological processes (Figure 2B). Among the level 1 pathways, the ‘Metabolism’ category encompasses the most KOs (4008), significantly outnumbering the remaining pathways. In contrast, ‘Genetic information processing’ and ‘Organismal systems’ only comprise 706 and 692 KOs, respectively. Moreover, these KOs engage with 51 level 2 pathways and 478 level 3 pathways, while also intertwining with 1610 GO terms.

For each family and superfamily, GDPF calculated distribution data across 10 000 global microbial communities, as well as their relative abundance and occurrence frequencies in 17 typical environments such as soil, ocean and gut (Figure 2C). Almost all families display cross-environment distribution, with over 60% of families present in all 17 environments (Supplementary Figure S1).

### Data browsing

**Figure 3.**
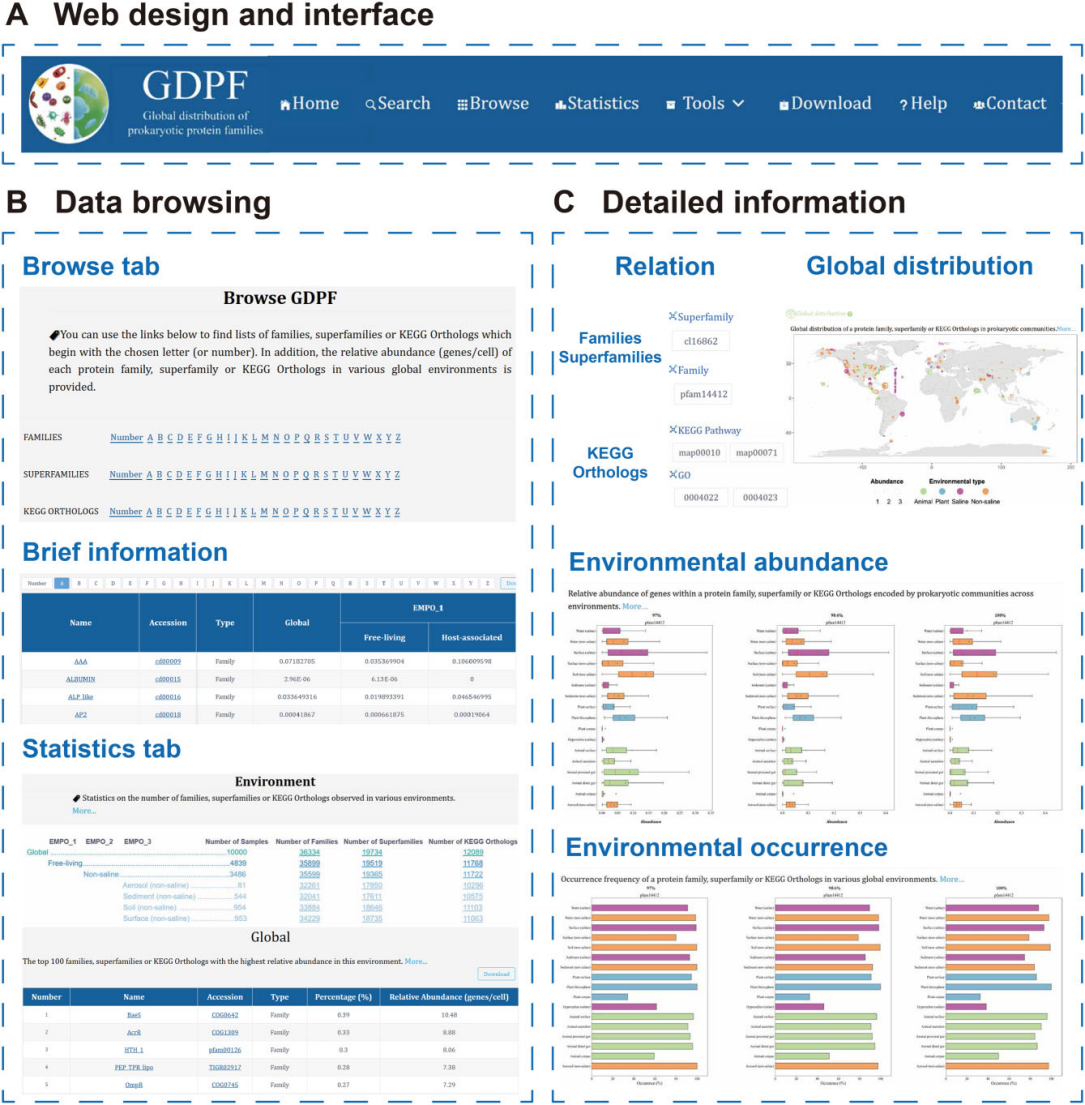
Browse in the GDPF database. (**A**) Website design and interface. (**B**) Data browsing interface. (**C**) Detailed information interface.

The GDPF database offers a user-friendly web interface to comprehensively display distribution data of different families and superfamilies within the global microbial communities **(**Figure 3A). The ‘Browse’ tab provides a browsing method based on different family categorizations and alphabetical sorting of the first letters, and upon clicking on either ‘Number’ or ‘A–Z’, users are redirected to the corresponding ‘Brief information’ interface, where each family or superfamily is shown as an independent entry (Figure 3B). Each entry contains the following information: the name, accession, type and relative abundance in the EMPO hierarchical environment (using 97% identity threshold). Users can adjust the number of entries displayed on each interface, and the entries are arranged with an alternately dark or light background. Mouseover provides the corresponding explanation for each table heading, along with a description for each entry. Users can click the ‘Download’ button to quickly obtain the family distribution data in the table. Upon clicking the name or accession of each entry, users are redirected to that family or superfamily’s ‘Detailed information’ interface.

The ‘Detailed information’ interface comprises five modules, providing global distribution information for each family or superfamily (Figure 3C). The description module offers the name, accession and a brief functional introduction of the entry, accompanied by links for navigating to its source database and InterPro database (if available) interfaces (32). The relation module provides the association information with families and superfamilies, and for KOs, it offers associations with orthologs and KEGG pathways, as well as GO terms. The global distribution module illustrates the distribution of the entry across the global biosphere, plotting data based on latitude and longitude coordinates from 10 000 EMP samples. Each plotted point corresponds to a sample, with circle size indicating its relative abundance and color defined by EMPO_2. The environmental abundance module showcases the variations in relative abundances of the entry in 17 typical EMPO_3 environments, utilizing a box plot for the statistical representation of each environment. This module provides data comparisons based on three thresholds (>97%, 98.6% or 100% 16S rRNA identities), and clicking on the images yields corresponding statistical data tables. The environmental occurrence module presents the differences in occurrence frequencies of the entry across EMPO_3 environments using column charts, also based on the three thresholds. Finally, in the ‘Detailed information’ interface, clicking ‘Download’ allows users to download all raw data and related high-resolution images of the entry in 10 000 microbial communities.

The ‘Statistics’ tab displays the number of functional families detected in different environments through EMPO hierarchical classification. Mouseover provides an interactive map that displays sampling locations within each EMPO_3 environment. By clicking on links, users can access information on the top 100 families, superfamilies or KOs ranked by abundance in specific environments. Clicking on the name or accession of each entry directs users to the corresponding ‘Detailed information’ interface.

### Data search

**Figure 4.**
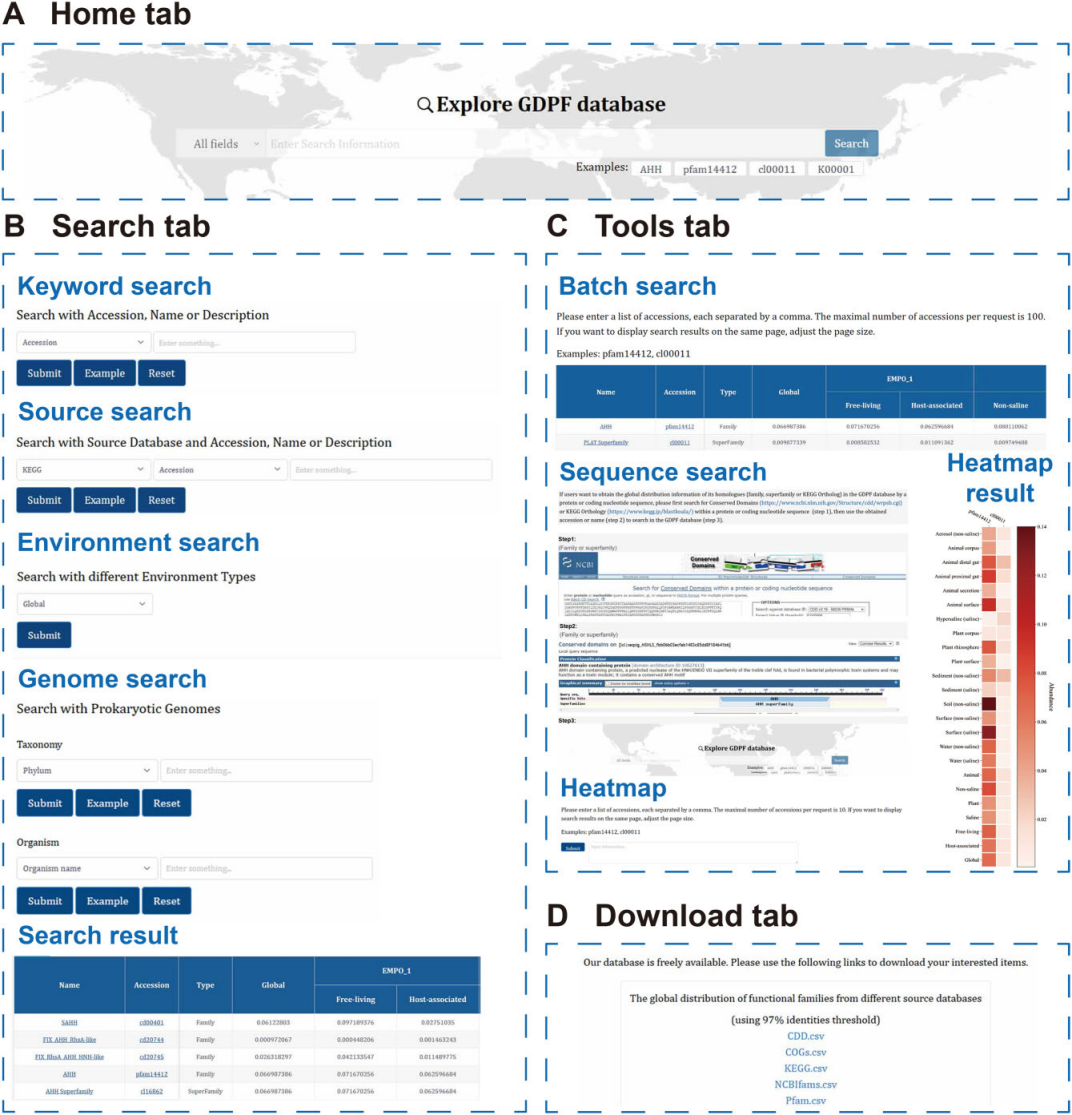
Data search and expanded analysis tools in the database. (**A**) Search box in the ‘Home’ tab. (**B**) ‘Search’ tab. (**C**) ‘Tools’ tab. (**D**) ‘Download’ tab.

Users can conveniently query global geographical distribution information for specific entries using the search box in the ‘Home’ tab (Figure 4A). This search box allows fuzzy searches based on the entry accession or name. The ‘Search’ tab offers a more powerful search engine including four searching methods to help users accurately find the entries of interest in the database (Figure 4B). Keyword search enables fuzzy searches using keywords for entry accession, name or description. Source search offers a drop-down filter option to filter the source database of entries. Environment search allows users to filter entries based on the EMPO hierarchical environments. Genome search empowers users to retrieve entry information within specific genomes, either through taxonomic classification or through genome accession. All the above search results are presented on the ‘Brief information’ interface, where they can be further clicked into the ‘Detailed information’ interface for each individual entry.

### Expanded tools and data download

The ‘Tools’ tab provides extended tools for batch retrieval of entries, annotating specific sequences into existing entries and generating heatmaps for comparing the relative abundances of different entries across various environments (Figure 4C). The ‘Batch search’ tool supports batch exact searches using accessions and users simply need to submit a comma-separated list of accessions. All the search results are presented on the ‘Brief information’ interface and further clicked to enter the ‘Detailed information’ interface for each entry. If users want to inquire about the global distribution patterns of homologous families of a specific protein, they can employ RPS-BLAST or BlastKOALA to annotate the sequence, acquiring the corresponding family accession or name for subsequent retrieval within the GDPF database. The ‘Heatmap’ tool provides an online platform to visually contrast distribution disparities of different entries across diverse environments. Users can submit a maximum of 10 comma-separated accession lists, upon which GDPF will depict a distribution heatmap reflecting the relative abundance of different entries in distinct environments. Users can download the heatmap and its original data as needed.

Batch downloading of GDPF database entries is available in the ‘Download’ tab, where all entries are categorized and packaged based on their source databases for easy user extraction (Figure 4D). In the ‘Help’ tab, users can access the introduction, technical route and usage guidelines of the GDPF database. For any inquiries related to website usage and development, users can effortlessly get in touch with us through the contact details provided in the ‘Contact’ tab.

## Summary and future perspectives

Microbial communities are a crucial component of Earth’s ecosystems, participating in all essential ecological processes through diverse functional genes (4). Functional diversity is increasingly recognized by microbiologists as a pivotal link between patterns of biodiversity and ecosystem functioning, determining trophic relationships and interactions among microorganisms, their participation in biogeochemical cycles and their response to environmental change (33–35). The GDPF database provides a unique, readily explorable archive of information on the distribution of microbial functional families from a global perspective. This resource not only summarizes a wide array of widely used functional family classifications but also presents data showcasing the distribution of families across environments. Moreover, it equips researchers with heatmap tools to effectively contrast the discrepancies in the distribution of multiple families across various environments. The GDPF resource has been extensively employed to study the cross-environmental distribution patterns of an extensive array of microbial functions (22,36–38). Looking ahead, as the realm of high-throughput sequencing data continues its rapid expansion, we are committed to routinely updating the environmental distribution data pertaining to microbial functional families. This endeavor is driven by the aim of catering to the global user community, ensuring that the GDPF database remains an invaluable asset for uncovering the critical relationships between functional families and the environments that span the globe.

## Supplementary Material

gkad869_Supplemental_File

## Data Availability

The GDPF database is freely available to the public without registration or login requirements (http://bioinfo.qd.sdu.edu.cn/GDPF/). The Python and R code used for the analyses and database construction are available online at https://github.com/panzhuoddv/Database and https://doi.org/10.5281/zenodo.8376882.

## References

[1] Thompson L.R. , Sanders J.G., McDonald D., Amir A., Ladau J., Locey K.J., Prill R.J., Tripathi A., Gibbons S.M., Ackermann G. et al. A communal catalogue reveals Earth’s multiscale microbial diversity. Nature. 2017; 551:457–463. 29088705 10.1038/nature24621 PMC6192678

[2] Hug L.A. , Baker B.J., Anantharaman K., Brown C.T., Probst A.J., Castelle C.J., Butterfield C.N., Hernsdorf A.W., Amano Y., Ise K. et al. A new view of the tree of life. Nat. Microbiol. 2016; 1:16048. 27572647 10.1038/nmicrobiol.2016.48

[3] Fierer N. Embracing the unknown: disentangling the complexities of the soil microbiome. Nat. Rev. Microbiol. 2017; 15:579–590. 28824177 10.1038/nrmicro.2017.87

[4] Falkowski P.G. , Fenchel T., Delong E.F. The microbial engines that drive Earth’s biogeochemical cycles. Science. 2008; 320:1034–1039. 18497287 10.1126/science.1153213

[5] Escalas A. , Paula F.S., Guilhaumon F., Yuan M.T., Yang Y.F., Wu L.W., Liu F.F., Feng J.E., Zhang Y.G., Zhou J.Z. Macroecological distributions of gene variants highlight the functional organization of soil microbial systems. ISME J. 2022; 16:726–737. 34580430 10.1038/s41396-021-01120-8 PMC8857198

[6] Coelho L.P. , Alves R., del Rio A.R., Myers P.N., Cantalapiedra C.P., Giner-Lamia J., Schmidt T.S., Mende D.R., Orakov A., Letunic I. et al. Towards the biogeography of prokaryotic genes. Nature. 2022; 601:252–256. 34912116 10.1038/s41586-021-04233-4 PMC7613196

[7] Louca S. , Polz M.F., Mazel F., Albright M.B.N., Huber J.A., O’Connor M.I., Ackermann M., Hahn A.S., Srivastava D.S., Crowe S.A. et al. Function and functional redundancy in microbial systems. Nat. Ecol. Evol. 2018; 2:936–943. 29662222 10.1038/s41559-018-0519-1

[8] Galperin M.Y. , Wolf Y.I., Makarova K.S., Alvarez R.V., Landsman D., Koonin E.V. COG database update: focus on microbial diversity, model organisms, and widespread pathogens. Nucleic Acids Res. 2021; 49:D274–D281. 33167031 10.1093/nar/gkaa1018 PMC7778934

[9] Kanehisa M. , Furumichi M., Sato Y., Ishiguro-Watanabe M., Tanabe M. KEGG: integrating viruses and cellular organisms. Nucleic Acids Res. 2021; 49:D545–D551. 33125081 10.1093/nar/gkaa970 PMC7779016

[10] Mistry J. , Chuguransky S., Williams L., Qureshi M., Salazar G.A., Sonnhammer E.L.L., Tosatto S.C.E., Paladin L., Raj S., Richardson L.J. et al. Pfam: the protein families database in 2021. Nucleic Acids Res. 2021; 49:D412–D419. 33125078 10.1093/nar/gkaa913 PMC7779014

[11] Wang J. , Chitsaz F., Derbyshire M.K., Gonzales N.R., Gwadz M., Lu S., Marchler G.H., Song J.S., Thanki N., Yamashita R.A. et al. The Conserved Domain Database in 2023. Nucleic Acids Res. 2023; 51:D384–D388. 36477806 10.1093/nar/gkac1096 PMC9825596

[12] Sunagawa S. , Coelho L.P., Chaffron S., Kultima J.R., Labadie K., Salazar G., Djahanschiri B., Zeller G., Mende D.R., Alberti A. et al. Structure and function of the global ocean microbiome. Science. 2015; 348:1261359. 25999513 10.1126/science.1261359

[13] Bahram M. , Hildebrand F., Forslund S.K., Anderson J.L., Soudzilovskaia N.A., Bodegom P.M., Bengtsson-Palme J., Anslan S., Coelho L.P., Harend H. et al. Structure and function of the global topsoil microbiome. Nature. 2018; 560:233–237. 30069051 10.1038/s41586-018-0386-6

[14] Zou Y.Q. , Xue W.B., Luo G.W., Deng Z.Q., Qin P.P., Guo R.J., Sun H.P., Xia Y., Liang S.S., Dai Y. et al. 1,520 reference genomes from cultivated human gut bacteria enable functional microbiome analyses. Nat. Biotechnol. 2019; 37:179–185. 30718868 10.1038/s41587-018-0008-8 PMC6784896

[15] Li W.X. , Liang H.W., Lin X.Q., Hu T.Y., Wu Z.N., He W.X., Wang M.M., Zhang J.H., Jie Z.Y., Jin X. et al. A catalog of bacterial reference genomes from cultivated human oral bacteria. npj Biofilms Microbiomes. 2023; 9:45. 37400465 10.1038/s41522-023-00414-3 PMC10318035

[16] Lin X. , Hu T., Chen J., Liang H., Zhou J., Wu Z., Ye C., Jin X., Xu X., Zhang W. The genomic landscape of reference genomes of cultivated human gut bacteria. Nat. Commun. 2023; 14:1663. 36966151 10.1038/s41467-023-37396-x PMC10039858

[17] Zhang Z. , Wang J.N., Wang J.L., Wang J.J., Li Y.Z. Estimate of the sequenced proportion of the global prokaryotic genome. Microbiome. 2020; 8:134. 32938501 10.1186/s40168-020-00903-z PMC7496214

[18] Garcia-Garcera M. , Rocha E.P. Community diversity and habitat structure shape the repertoire of extracellular proteins in bacteria. Nat. Commun. 2020; 11:758. 32029728 10.1038/s41467-020-14572-x PMC7005277

[19] Machado D. , Maistrenko O.M., Andrejev S., Kim Y., Bork P., Patil K.R., Patil K.R. Polarization of microbial communities between competitive and cooperative metabolism. Nat. Ecol. Evol. 2021; 5:195–203. 33398106 10.1038/s41559-020-01353-4 PMC7610595

[20] Douglas G.M. , Maffei V.J., Zaneveld J.R., Yurgel S.N., Brown J.R., Taylor C.M., Huttenhower C., Langille M.G. PICRUSt2 for prediction of metagenome functions. Nat. Biotechnol. 2020; 38:685–688. 32483366 10.1038/s41587-020-0548-6 PMC7365738

[21] Zhang Y.L. , Wang Y.L., Tang M.X., Zhou J.Z., Zhang T. The microbial dark matter and “wanted list” in worldwide wastewater treatment plants. Microbiome. 2023; 11:59. 36973807 10.1186/s40168-023-01503-3 PMC10045942

[22] Liu Y. , Liu S., Pan Z., Ren Y., Jiang Y.R., Wang F., Li D.D., Li Y.Z., Zhang Z. PAT: a comprehensive database of prokaryotic antimicrobial toxins. Nucleic Acids Res. 2023; 51:D452–D459. 36243963 10.1093/nar/gkac879 PMC9825508

[23] Amir A. , McDonald D., Navas-Molina J.A., Kopylova E., Morton J.T., Xu Z.Z., Kightley E.P., Thompson L.R., Hyde E.R., Gonzalez A. et al. Deblur rapidly resolves single-nucleotide community sequence patterns. mSystems. 2017; 2:e00191-16. 28289731 10.1128/mSystems.00191-16 PMC5340863

[24] Li W.J. , O’Neill K.R., Haft D.H., DiCuccio M., Chetvernin V., Badretdin A., Coulouris G., Chitsaz F., Derbyshire M.K., Durkin A.S. et al. RefSeq: expanding the Prokaryotic Genome Annotation Pipeline reach with protein family model curation. Nucleic Acids Res. 2021; 49:D1020–D1028. 33270901 10.1093/nar/gkaa1105 PMC7779008

[25] Chun J. , Oren A., Ventosa A., Christensen H., Arahal D.R., da Costa M.S., Rooney A.P., Yi H., Xu X.W., De Meyer S. et al. Proposed minimal standards for the use of genome data for the taxonomy of prokaryotes. Int. J. Syst. Evol. Microbiol. 2018; 68:461–466. 29292687 10.1099/ijsem.0.002516

[26] Kanehisa M. , Sato Y., Morishima K. BlastKOALA and GhostKOALA: KEGG tools for functional characterization of genome and metagenome sequences. J. Mol. Biol. 2016; 428:726–731. 26585406 10.1016/j.jmb.2015.11.006

[27] Hernandez-Plaza A. , Szklarczyk D., Botas J., Cantalapiedra C.P., Giner-Lamia J., Mende D.R., Kirsch R., Rattei T., Letunic I., Jensen L.J. et al. eggNOG 6.0: enabling comparative genomics across 12 535 organisms. Nucleic Acids Res. 2023; 51:D389–D394. 36399505 10.1093/nar/gkac1022 PMC9825578

[28] Aleksander S.A. , Balhoff J., Carbon S., Cherry J.M., Drabkin H.J., Ebert D., Feuermann M., Gaudet P., Harris N.L., Hill D.P. et al. The Gene Ontology knowledgebase in 2023. Genetics. 2023; 224:iyad031. 36866529 10.1093/genetics/iyad031 PMC10158837

[29] Letunic I. , Khedkar S., Bork P. SMART: recent updates, new developments and status in 2020. Nucleic Acids Res. 2021; 49:D458–D460. 33104802 10.1093/nar/gkaa937 PMC7778883

[30] Klimke W. , Agarwala R., Badretdin A., Chetvernin S., Ciufo S., Fedorov B., Kiryutin B., O’Neill K., Resch W., Resenchuk S. et al. The National Center for Biotechnology Information’s Protein Clusters Database. Nucleic Acids Res. 2009; 37:D216–D223. 18940865 10.1093/nar/gkn734 PMC2686591

[31] Haft D.H. , Selengut J.D., Richter R.A., Harkins D., Basu M.K., Beck E. TIGRFAMs and genome properties in 2013. Nucleic Acids Res. 2013; 41:D387–D395. 23197656 10.1093/nar/gks1234 PMC3531188

[32] Paysan-Lafosse T. , Blum M., Chuguransky S., Grego T., Pinto B.L., Salazar G.A., Bileschi M.L., Bork P., Bridge A., Colwell L. et al. InterPro in 2022. Nucleic Acids Res. 2023; 51:D418–D427. 36350672 10.1093/nar/gkac993 PMC9825450

[33] Loreau M. , Naeem S., Inchausti P., Bengtsson J., Grime J.P., Hector A., Hooper D.U., Huston M.A., Raffaelli D., Schmid B. et al. Biodiversity and ecosystem functioning: current knowledge and future challenges. Science. 2001; 294:804–808. 11679658 10.1126/science.1064088

[34] Bardgett R.D. , van der Putten W.H. Belowground biodiversity and ecosystem functioning. Nature. 2014; 515:505–511. 25428498 10.1038/nature13855

[35] Lamarque P. , Lavorel S., Mouchet M., Quetier F. Plant trait-based models identify direct and indirect effects of climate change on bundles of grassland ecosystem services. Proc. Natl Acad. Sci. U.S.A. 2014; 111:13751–13756. 25225382 10.1073/pnas.1216051111 PMC4183316

[36] Li D.D. , Wang J.L., Liu Y., Li Y.Z., Zhang Z. Expanded analyses of the functional correlations within structural classifications of glycoside hydrolases. Comput. Struct. Biotechnol. J. 2021; 19:5931–5942. 34849197 10.1016/j.csbj.2021.10.039 PMC8602953

[37] Zhang Z. , Liu Y., Zhang P., Wang J.N., Li D.D., Li Y.Z. Proteins are versatile clips that enrich the antimicrobial weapon arsenals of prokaryotes. mSystems. 2021; 6:e00953-21. 34874775 10.1128/mSystems.00953-21 PMC8651086

[38] Li D.D. , Zhang Z., Wang J.N., Zhang P., Liu Y., Li Y.Z. Estimate of the degradation potentials of cellulose, xylan, and chitin across global prokaryotic communities. Environ. Microbiol. 2023; 25:397–409. 36446618 10.1111/1462-2920.16290

