# Supplementary material for: GDPF: a data resource for the distribution of prokaryotic protein families across the global biosphere
Source: Nucleic Acids Res. 2023 Oct 12;52(D1):D724–31. doi: 10.1093/nar/gkad869 (PMC10767866; doi:10.1093/nar/gkad869)
Supplement: gkad869_Supplemental_File [file gkad869_supplemental_file.pdf]

Supplementary Materials for

GDPF: a data resource for the distribution of prokaryotic protein families across the global biosphere

Zhuo Pan[1, †], Dan-dan Li[2, †], Peng Li[1], Yu Geng[1], Yiru Jiang[1], Ya Liu[1, 3], Yue-zhong Li[1, *], Zheng Zhang[1, *]

[1] *State Key Laboratory of Microbial Technology, Institute of Microbial Technology, Shandong University, Qingdao 266237, China*

[2] *Institute of Marine Science and Technology, Shandong University, Qingdao 266237, China*

[3] *Suzhou Research Institute, Shandong University, Suzhou 215123, China*

Content

Supplementary Figure S1 and Table S1.

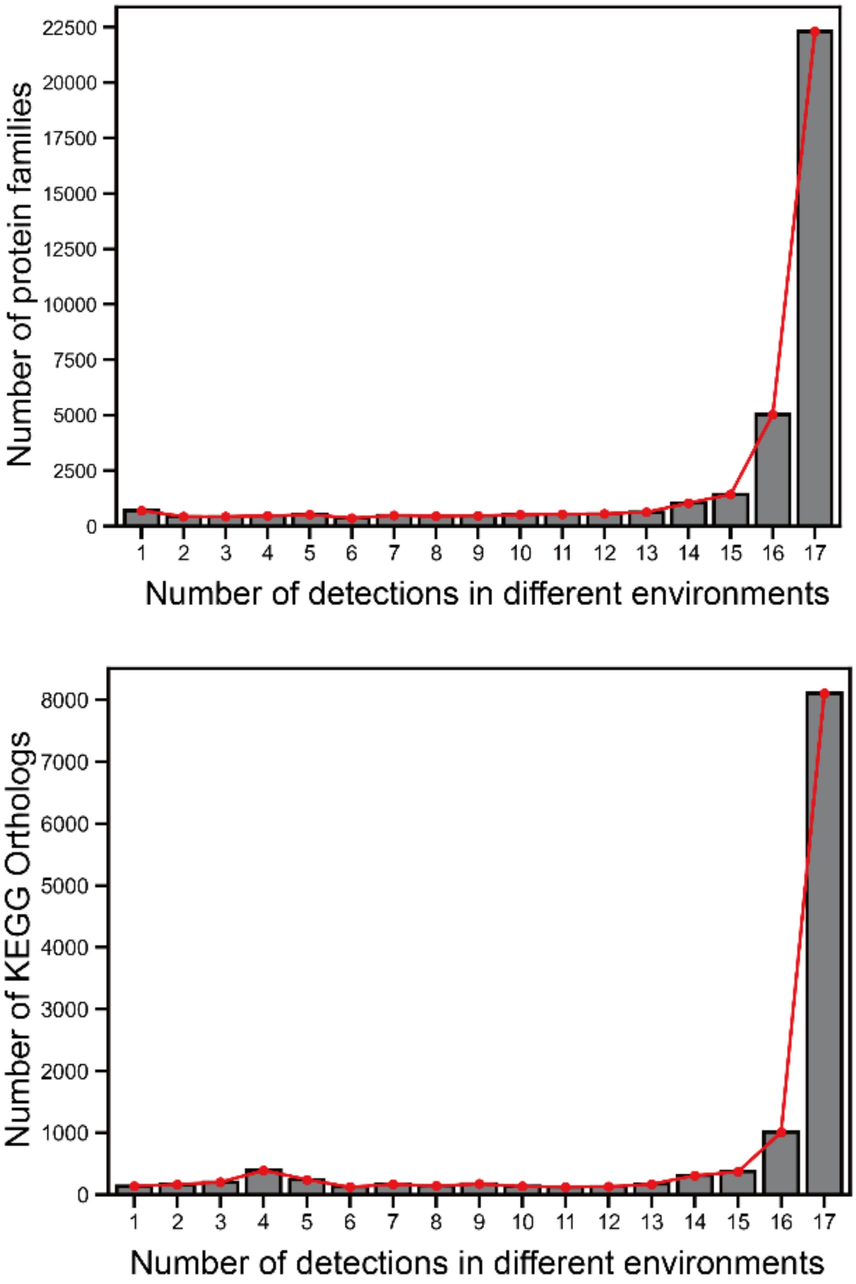

Supplementary Figure S1. The distribution of KOs or protein families across EMPO_3 level environments.

Supplementary Table S1. The EMPO environmental classification systems.

EMPO_1	EMPO_2	EMPO_3	Examples	Samples
Free-living	Non-saline	Aerosol (non-saline)	aerosolized dust or liquid	81
Free-living	Non-saline	Sediment (non-saline)	sediment from lake, pond, river (<5 psu)	544
Free-living	Non-saline	Soil (non-saline)	soil from forest, grassland, tundra, desert, etc.	954
Free-living	Non-saline	Surface (non-saline)	biofilm from wet (<5 psu) or dry surface, wood, dust, microbial mat	953
Free-living	Non-saline	Water (non-saline)	fresh water from lake, pond, river (<5 psu)	954
Free-living	Saline	Hypersaline (saline)	water from hypersaline sample or brine (>50 psu)	13
Free-living	Saline	Sediment (saline)	sediment from ocean, sea, estuary, mangrove, beach (>5 psu)	541
Free-living	Saline	Surface (saline)	biofilm from wet or underwater surface or microbial mat (>5 psu)	117
Free-living	Saline	Water (saline)	salt water from ocean, sea, estuary, mangrove, coral reef (>5 psu)	682
Host-associated	Animal	Animal corpus	tissue of sponge, coral, gill, siphon, carcass, etc., or whole small animal	322
Host-associated	Animal	Animal distal gut	feces, stool	953
Host-associated	Animal	Animal proximal gut	gut intestine, gizzard, crop, lumen, mucosa	354
Host-associated	Animal	Animal secretion	saliva, breast milk, vaginal secretion	917
Host-associated	Animal	Animal surface	skin, sebum, mucus, slime	987
Host-associated	Plant	Plant corpus	tissue of leaf, stem, fruit, algae	123
Host-associated	Plant	Plant rhizosphere	plant root system, may include some soil	552
Host-associated	Plant	Plant surface	leaf or kelp surface biofilm	953
